# Migraine aura-like symptoms at onset of stroke and stroke-like symptoms in migraine with aura

**DOI:** 10.3389/fneur.2022.1004058

**Published:** 2022-09-14

**Authors:** Adrian Scutelnic, Lukas A. Kreis, Morin Beyeler, Mirjam R. Heldner, Thomas R. Meinel, Johannes Kaesmacher, Arsany Hakim, Marcel Arnold, Urs Fischer, Heinrich P. Mattle, Christoph J. Schankin, Simon Jung

**Affiliations:** ^1^Department of Neurology, Inselspital, University Hospital Bern, University of Bern, Bern, Switzerland; ^2^Institute of Diagnostic and Interventional Neuroradiology, Inselspital, University Hospital and University of Bern, Bern, Switzerland; ^3^Department of Neurology and Stroke Centre, University Hospital Bern, University of Bern, Basel, Switzerland

**Keywords:** migraine aura, ischemic stroke, spreading depolarization, differential diagnosis, clinical

## Abstract

**Background and objectives:**

In general, suddenly occurring neurological deficits, i.e., negative neurological symptoms, are considered symptoms of focal cerebral ischemia, while positive irritative symptoms with gradual onset are viewed as the characteristics of migraine aura. Nevertheless, cortical spreading depolarization, the pathophysiological basis of migraine aura, has also been observed in acute ischemic stroke. The aim of our study was to determine the frequency of migraine aura-like symptoms at ischemic stroke onset and stroke-like symptoms in migraine with aura.

**Methods:**

We interviewed 350 consecutive patients with ischemic stroke and 343 with migraine with aura using a structured questionnaire. Stroke diagnosis was confirmed by imaging, and migraine with aura was diagnosed according to the current criteria of the International Headache Society. Patients with wake-up strokes or severe cognitive deficits that precluded a useful interview were excluded from the study.

**Results:**

Seventy-eight patients with stroke (22.3%) reported visual symptoms, 145 (41.4%) sensory symptoms, 197 (56.3%) a paresis, and 201 patients (57.4%) more than one symptom, compared to 326 migraine patients with aura (95%) with visual symptoms (*P* < 0.001), 175 (51%) with sensory symptoms (*p* = 0.011), 50 (14.6%) with paresis (*P* < 0.001), and 211 (61.5%) with more than one symptom (*p* = 0.27). Among patients with stroke, migraine-like symptoms were frequent: 36 patients (46.2%) with visual disturbance and 78 (53.8%) with sensory symptoms experienced irritative sensations. Paresis-onset in stroke lasted longer than 5 min in 43 patients (21.8%). Spreading of sensory and motor symptoms occurred in 37 (25.5%) and 37 (18.8%) patients, respectively. Stroke-like negative symptoms in migraine with aura occurred in 39 patients (12%) with visual symptoms, in 55 (31.4%) with sensory symptoms, and paresis appeared suddenly in 14 patients (28%). More than one symptom in succession occurred in 117 patients with stroke (58.2%) and in 201 migraine with aura patients (95.3%; *P* < 0.001).

**Conclusion:**

Many patients with stroke experience migraine-like symptoms at stroke onset, and many migraine with aura patients have stroke-like symptoms. Though overall the symptom frequencies of the two groups are significantly different, clarifying the differential diagnosis in an individual patient requires additional history elements, physical findings, or results of ancillary investigations.

## Background

There is a consensus that positive irritative neurological symptoms such as paresthesia or scintillations or gradual onset of visual, sensory, or speech disorders are considered characteristic expressions of migraine aura (1). Conversely, symptoms of deficit type such as hemianopsia, numbness, or sudden onset of deficits are thought to be characteristic of focal cerebral ischemia (2).

Cortical spreading depolarization, the pathophysiological mechanism underlying migraine aura, has also been recorded in other brain disorders including the ischemic penumbra (3, 4). Therefore, it is not surprising that migraine aura-like symptoms have been reported in ischemic stroke (5, 6). C.M. Fisher, in his seminal publication on “Migraine Accompaniments vs. Arteriosclerotic Ischemia” more than half a century ago, drew our attention to the interpretation of neurological spells (6). He pointed out that the differentiation of arteriosclerotic ischemic attacks from migraine accompaniments may pose a problem. This has clinical implications, since at the bedside it is very important but often difficult to distinguish migraine aura from acute ischemic stroke. Today, however, we do not know much more. We do not know the frequency of migraine-like symptoms at the onset of acute ischemic stroke. We still struggle to differentiate focal cerebral ischemia at onset and migraine aura. This prompted our study to compare clinical symptoms of patients with proven ischemic stroke and migraine patients with aura (MwA) and to assess the frequency of migraine aura-like symptoms in patients with ischemic stroke and stroke-like symptoms in patients with MwA.

## Methods

This is a single-center, observational study. Consecutive patients with acute ischemic stroke confirmed by CT or MRI imaging, and patients with MwA diagnosed according to the International Classification of Headache Disorders, third edition (ICHD-3) (1) were recruited between 03/2019 and 08/2021, patients with stroke mostly from our stroke center and patients with migraine from the outpatient headache clinic. The patients were interviewed in German, French, or English using a structured questionnaire (Supplemental File), either at the bedside or by phone by neurology residents (AS, MB) or a medical student (LAK) who had been specially trained to perform such interviews. There were multiple answer choices for every question (e.g., type of sensory disturbance), and the participant had to choose a suitable answer for their own symptoms. The questions were asked as close as possible, listing the different answer possibilities. If the participant found no answer appropriate to describe his or her symptoms, the participant could also give an open answer using his or her own terms.

### Definitions

Positive visual symptoms were defined as scintillations, scintillating scotoma, zigzag lines, bright vision, colored vision, or visual hallucinations and considered migraine-like (1). Visual symptoms moving over the visual field were also considered migraine-like. Negative visual symptoms were defined as dark or gray vision, isolated blurry vision and partial or complete blindness, or both and considered stroke-like.

Positive sensory symptoms were defined as sensations of tingling, pins and needles or both and considered migraine-like. Spreading of symptoms was also regarded as migraine-like.

Numbness was defined as a negative sensory symptom and considered stroke-like.

Weakness was defined as a negative symptom and stroke-like except for motor symptoms with onset lasting 5 min or longer or when weakness was spreading gradually from one body part to another.

Speech disorders were classified as fluent, non-fluent, or mixed aphasia or dysarthria only. They could not be categorized as positive or negative symptoms and were included only in the analysis of multiple symptoms.

### Inclusion and exclusion criteria

The inclusion criteria for participants with stroke were: (a) confirmed diagnosis of ischemic brain infarction with imaging according to the 2013 AHA/ASA definition (7), (b) stroke within 6 months prior to study inclusion, and (c) age >18 years. The inclusion criteria for the participants with MwA were: (a) diagnosis of definite MwA according to ICHD-3 (1), (b) last migraine attack with aura within 12 months prior to study inclusion, and (c) age > 18 years. Exclusion criteria were (a) severe cognitive deficits that precluded a useful interview and (b) wake-up strokes.

### Data analysis

The statistical analysis was performed in STATA/MP 16.0, StataCorp LLC. Descriptive statistics to compare the groups were used. Categorical data are presented in counts, continuous data as means, and interquartile ranges. Categorical variables were compared using the χ^2^ test or Fisher's exact test, as appropriate and continuous variables using the Mann–Whitney U test. For comparison of three or more groups, the Bonferroni correction has been performed.

### Ethics

The study has been approved by the local ethics committee (KEK 2018-02258). Informed consent has been obtained from all participants.

### Data availability statement

Anonymized data will be made available by reasonable request.

## Results

Four hundred thirty-one patients with ischemic stroke and adequate cognition for interviews and 343 patients with migraine were interviewed. Eighty-one patients with stroke were excluded, since they had symptoms at wake-up, leaving 350 patients with ischemic stroke for analysis. The baseline characteristics of patients with migraine and stroke are shown in Table 1.

**Table 1:** Baseline characteristics and risk factors for stroke in migraine with aura and stroke groups.

**Baseline**	**Stroke**	**Migraine with aura**	***p*-value**
Number of patients	350	343	
Age (median, IQR)	71, 62–78	29, 28–52	<0.001
Number of women (n; %, f/m)	39	69	<0.001
Days from index event or last aura attack to interview (median, IQR)	2, 1–3	28, 7–118	<0.001
NIHSS, median, IQR (stroke only)	1, 0–3		
**Risk factors**
Active smoking n (%)	121 (34.6)	103 (30)	0.2
Illicit drug use n (%)	4 (1.1)	14 (4.1)	0.015
High blood pressure n (%)	233 (66.6)	64 (18.7)	<0.001
Dyslipidemia n (%)	305 (87.4)	29 (8.5)	<0.001
Diabetes mellitus n (%)	78 (22.3)	8 (2.3)	<0.001
Sleep apnea n (%)	162 (46.3)	14 (4.1)	<0.001
Family history of stroke or myocardial infarction n (%)	94 (26.9)	76 (22.2)	0.15
Chronic kidney failure n (%)	42 (12)	1 (0.3)	<0.001
Contraceptive hormonal treatment n (%)	5 (3.6)	36 (15.3)	<0.001
Atrial fibrillation n (%)	44 (12.6)	3 (0.9)	<0.001
Chronic inflammatory disease or active tumor n (%)	47 (13.4)	26 (7.6)	0.012
**Co-morbidities**
Migraine with aura (stroke only) n (%)	15 (4)		
History of ischemic stroke (migraine only) n (%)		21 (6.1)	
Epilepsy n (%)	1 (0.3)	7 (2)	0.03
Seizure at presentation (stroke only) n (%)	2 (0.6)		
**Acute treatment (stroke only)**
Conservative* n (%)	221 (63.1)		
IVT or IAT n (%)	129 (36.9)		
IAT only n (%)	28 (8)		
IVT only n (%)	81 (23.1)		
IVT and IVT n (%)	20 (5.7)		

IQR, interquartile range; NIHSS, National Institutes of Health Stroke Scale; IAT, intra-arterial therapy; IVT, intravenous thrombolysis, *no IAT or IVT.

Patients with stroke were older (median 71 years, IQR 62–78) compared to patients with migraine (median 29 years, IQR 28–52). There were more men in the stroke group (61% men), and more women in the MwA group (69% women). The median NIHSS score of patients with stroke was 1 (IQR 0–3) and in 313 patients (89.4%) ≤ 4. Patients in the stroke group were interviewed for a median of 2 days (IQR 1–3) after stroke onset, and patients with migraine a median of 28 days (IQR 7–118) after the last MwA attack.

**Figure 1:**
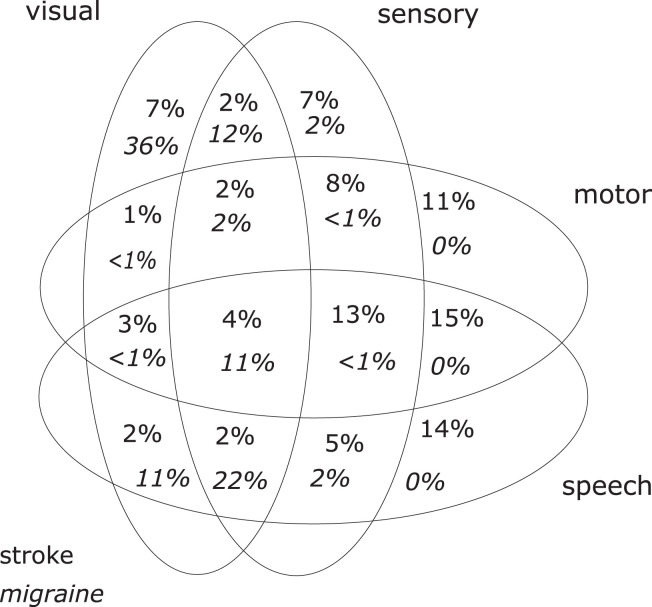
Distribution of symptoms in ischemic stroke (*n* = 350) and migraine with aura (*n* = 343).

Table 2 summarizes the main findings and Table 3 shows the headache characteristics of both groups. The distributions of symptoms are shown in Figure 1.

**Table 2:** Symptoms in stroke and MwA patients.

		**Stroke patients**		**MwA patients**		***P*-value** ** (Chi-square)**	***P*-value for multiple comparisons (Bonferroni)**
		350		343			
**Visual symptoms**		**78 (22.3%)**		**326 (95.0%)**		***P** **<*** **0.001**	
	Positive irritative		25 (32.1%)		220 (67.5%)	*P < * 0.001	
	Positive and negative		11 (14.1%)		67 (20.6%)		*P =* 0.004
	Negative		42 (53.8%)		39 (12%)		
	Moving symptoms		16 (20.5%)		181 (55.5%)	*P < * 0.001	
**Sensory symptoms**		**145 (41.4%)**		**175 (51.0%)**		***P** **=*** **0.011**	
	Positive irritative		51 (35.2%)		43 (24.6%)	*P < * 0.001	
	Positive and negative		27 (18.6%)		77 (44%)		*P < * 0.001
	Negative		67 (46.2%)		55 (31.4%)		
	Spreading sensory symptoms		37 (25,5%)		106 (60.6%)	*P < * 0.001	
**Paresis**		**197 (56.3%)**		**50 (14.6%)**		***P** **<*** **0.001**	
	Sudden onset		116 (58.8%)		14 (28%)	*P < * 0.01	
	Onset < 60 s		16 (8.1%)		0		*P < * 0.001
	Onset > 5 min		43 (21.8%)		26 (52%)		
	Spreading motor symptoms		37 (18.8%)		22 (44%)	*P < * 0.001	
Multiple symptoms*		**201 (57.4%)**		**211 (61.5%)**		***P** **=*** **0.27**	
	Symptoms in succession		117 (58.2%)		201 (95.3%)	*P < * 0.001	

^*^Multiple symptoms include at least two of visual and sensory symptoms, paresis and aphasia or dysarthria.

**Table 3:** Headache characteristics of patients with acute ischemic stroke and migraine with aura.

	**Stroke** ** (*N* = 350)**	**Migraine with aura (*N* = 343)**
Headache n (%)	90 (26)	319 (93)
Quality
Pulsating n (%)	11 (3)	143 (42)
Throbbing n (%)	5 (1)	74 (22)
Shooting/Stabbing n (%)	25 (7)	124 (36)
Pressing n (%)	58 (17)	130 (38)
Burning n (%)	7 (2)	24 (7)
Aggravation upon movement n (%)	22 (6)	257 (75)
Nausea n (%)	26 (7)	221 (64)
Photophobia n (%)	17 (5)	282 (82)
Phonophobia n (%)	8 (2)	252 (74)

### Visual symptoms

**Figure 2:**
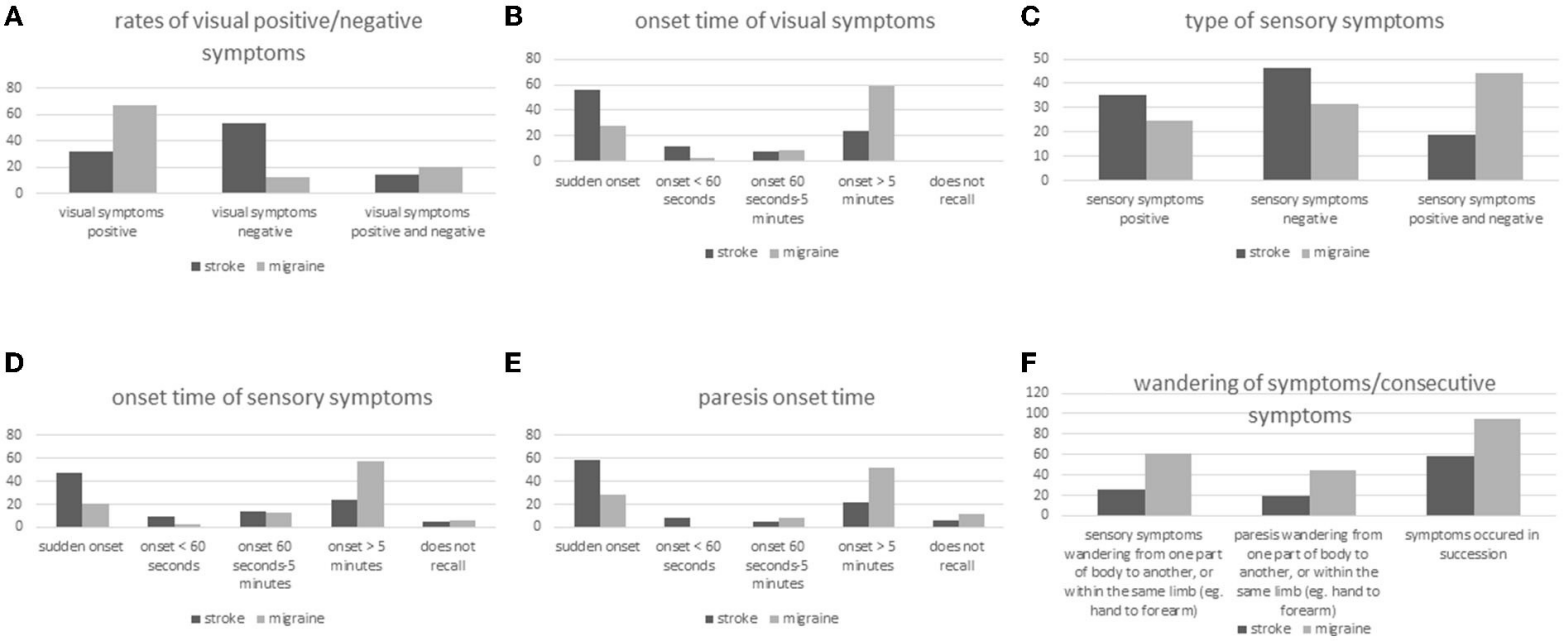
**(A–F)** Symptom type, symptom onset, and spreading of symptoms in stroke and migraine patients with aura.

Seventy-eight patients with stroke (22.3%) reported visual disturbances, compared to 326 patients with MwA (95%) (Figure 2A). Twenty-five of the 78 patients with stroke with visual disturbances (32.1%) had positive symptoms only, 11 (14.1%) had both positive and negative symptoms, and 42 (53.8%) had negative symptoms only. Among patients in the MwA group with visual disturbances (*n* = 326), 220 (67.5%) recorded positive symptoms only, 67 (20.6%) had both positive and negative symptoms, and 39 (12%) had negative symptoms only.

Visual disturbances appeared suddenly in 44 of the 78 patients with stroke (56.4%) and in 92 of the 326 patients with MwA (28.2%) (Figure 2B). Among the 78 stroke patients with visual disturbances, 9 (11.5%) reported onset of visual symptoms in < 60 s and among the 326 MwA patients 9 as well (2.8%). Visual symptoms developed gradually within > 5 min in 19 of the 78 patients with stroke (24.3%) and 193 of the 326 patients with MwA (59.2%). All patients with stroke remembered the onset time of visual disturbances, while four of the patients with MwA (1.2%) did not recall.

### Sensory symptoms

Among the 350 patients with stroke, 145 (41.4%) experienced sensory disturbances, compared to 175 (51%) of the 343 patients with migraine (Figure 2C). Fifty-one of the 145 stroke patients with sensory disturbance (35.2%) reported positive symptoms only, 27 (18.6%) both positive and negative symptoms and 67 (46.2%) negative symptoms only. Among MwA patients with sensory disturbance (*n* = 175), 43 (24.6%) remembered positive symptoms only, 77 (44%) both positive and negative symptoms, and 55 (31.4%) negative symptoms only.

Sensory disturbances appeared suddenly in 68 patients with stroke (46.9%) and 36 MwA patients (20.6%) (Figure 2D). Fourteen stroke patients (9.7%) and five patients with MwA (2.9%) reported onset of sensory disturbance in < 60 s and 35 (24.2%) and 101 (57.7%), respectively, onset duration >5 min. Eight patients with stroke (5.5%) and 10 patients with MwA (5.7%) did not recall the onset characteristics.

### Paresis

Paresis occurred in 197 (56.3%) patients with stroke and 50 patients with MwA (14.6%), among 116 patients with stroke (58.9%) and 14 patients with MwA (28%) suddenly, and among 16 (8.1%) patients with stroke in < 60 s (Figure 2E). None of the patients with MwA reported onset of paresis in < 60 s. However, 43 stroke patients (21.8%) and 26 MwA patients (52%) experienced 5 or more minutes of onset duration of paresis, while 13 (6.6%) and 6 (12%) did not recall the onset of paresis.

### Multiple symptoms and spreading of symptoms

Two hundred and one patients with stroke (57.4%) and 211 patients with MwA (61.5%) reported more than one symptom, i.e., more than only visual, sensory, motor, or speech disorders (Figure 2F). Symptoms occurred mostly sequentially and not simultaneously, namely among 117 (58.2%) patients with stroke and 201 patients with MwA (95.3%). Thirty-seven of 145 patients with stroke (25.5%) and 106/175 patients with MwA (60.6%) reported spreading of sensory symptoms from one part of the body to another, or within the same limb (e.g., from hand to forearm). Spreading of motor symptoms has been reported by 37/197 patients with stroke (18.8%) and 22/50 patients with MwA (44%). Sixteen patients with stroke (20%) and 181 patients with MwA (55%) reported visual symptoms moving over the visual field.

## Discussion

The major findings of our large series of patients with stroke diagnosed with modern neuroimaging and patients with MwA verified according to the ICHD-3 criteria are the high frequency of migraine-like positive irritative symptoms in ischemic stroke and the frequent occurrence of stroke-like negative symptoms in patients with MwA.

46.2% of patients with stroke with visual symptoms and 53.8% of the patients with sensory symptoms experienced migraine-like positive irritative symptoms. Furthermore, gradual symptom onset lasting more than 5 min occurred in 22.4% of stroke patients with visual symptoms, 24.2% with sensory symptoms, and 21.8% with paresis.

On the other hand, stroke-like negative symptoms were not uncommon in patients with MwA. 12% of patients with MwA with visual symptoms and 31.4% with sensory disturbances reported negative symptoms. Furthermore, 28.2% of those with visual symptoms, 20.6% of those with sensory symptoms, and 28% of those with paresis reported sudden symptom onset.

Our findings are in line with the report of Charles Miller Fisher in 1968 when he first pointed out the overlap of the clinical presentation of ischemic attacks and migraine aura (6). He found a considerable rate of positive symptoms with gradual onset in patients with stroke and negative symptoms with sudden onset in patients with migraine.

Positive symptoms with gradual onset in ischemic stroke may be explained by several pathomechanisms. First, cortical spreading depolarization, first described as cortical spreading depression (CSD) by Leão, is a wave of slowly propagating depolarization followed by neuronal silence within the brain tissue. CSD is considered the pathophysiological basis of migraine aura, but it is not specific (4). It has been detected in other conditions and fairly frequently in ischemic stroke (4, 8). In experimental animals such as mice, CSD can be induced by using microemboli without producing overt ischemia (9). Second, release phenomena of internally generated brain activity associated with positive symptoms caused by stroke lesions have been reported (10, 11). Third, clot growth and gradual obstruction of branching arterioles or gradual failing of collaterals could explain gradually increasing stroke symptoms, and forth, clot fragmentation and dislocation might cause the sequential onset of different symptoms and signs.

Unfortunately, negative symptoms with sudden onset have been increasingly interpreted as of ischemic origin and positive symptoms with gradual onset as non-ischemic. This is exemplified by the current development and validation of diagnostic criteria for transient ischemic attacks (TIA) that exclude positive symptoms with gradual onset from the clinical spectrum of TIA (2).

Comparing the symptoms of the stroke patient group and the MwA patient group, there is—as expected—a significant and unequivocal difference in the clinical presentation (Table 2). On the other hand, there is a large overlap between the clinical presentation of migraine aura and ischemic stroke (Figure 1), and this might bring the managing physician into a difficult position. The large overlap could make a clinical differentiation between the two conditions based on the history of symptoms alone difficult or even impossible in an individual patient.

Furthermore, patients with MwA are at increased risk of ischemic stroke. MwA doubles the risk of stroke, and especially in the young there is a strong association between MwA and stroke even in the absence of vascular risk factors (12, 13). Whether the doubling of stroke risk is due to primary MwA or due to a disorder causing secondary MwA and stroke is unknown (12, 14, 15). In addition, there is an association of MwA and patent foramen ovale (PFO), and in patients with cryptogenic stroke who have migraine, there is a high prevalence of PFO (79%) with right-to-left shunt (12, 16). This illustrates how different diseases can be hidden behind similar clinical phenotypes (17), and that the effective management of patients with stroke and MwA is often only possible with additional information on the patient's history, physical signs, and results of ancillary investigations.

On the other hand, negative symptoms in patients with MwA can indicate both a new attack with aura or an ischemic TIA or stroke. In the acute setting, this can also become a differential diagnostic problem, especially because strokes in patients with MwA rarely manifest as migrainous strokes (13, 18). Furthermore, the aura type can change from aura to aura (19).

Our study has several strengths. First, the results are based on a structured live interview, minimizing the chance of misinterpretation of the questions. Second, although the questions were asked in a closed manner, the participants could also provide an open answer, thus minimizing the risk of suggestion. Third, the interviews with patients with stroke were performed soon after the index event, thus minimizing the recall bias. Since patients with MwA experience similar auras frequently, the effect of recall bias is reduced. Fourth, the relatively large number of patients makes a statistically random result unlikely and allows for generalization.

Our study has also limitations. First, although the interviews were performed soon after the index stroke, the interviews of the patients with MwA were conducted later and we cannot exclude a recall bias. Second, we did not assess the cognitive status of our participants in detail and therefore cannot exclude falsely reported symptoms because of cognitive deficits. We included, however, only patients who were oriented, appeared to be cognitively normal in conversation, and with whom the interviewer could communicate without difficulties. Third, since the majority of included patients with stroke had a minor stroke (NIHSS ≤ 4 in 89.4%) the findings of this study might not be generalizable to patients with severe stroke. Forth, in patients with migraine with aura, we did not assess whether the aura had an intra-individual variability. Especially, we did not assess the consistency of symptoms from aura to aura. However, we assessed the most common aura, which might have mitigated this problem.

In conclusion, many patients with stroke experience migraine-like symptoms at stroke onset and many MwA patients stroke-like symptoms. Though overall the symptom frequencies of the two groups are significantly different, clarifying the differential diagnosis in an individual patient requires additional history elements, physical findings, or results of ancillary investigations.

## Data availability statement

Anonymized data will be made available by reasonable request by the corresponding author.

## Ethics statement

The studies involving human participants were reviewed and approved by Kantonale Ethikkommission Bern. The patients/participants provided their written informed consent to participate in this study.

## Author contributions

Study concept: AS, CS, and SJ. Data aquisition: AS, LK, and MB. Data analysis: AS, HPM, CS, and SJ. Drafting of the manuscript: AS, CS, SJ, and HPM. All authors contributed to critical revision of the article for intellectual content and approved the submitted version.
