# Erratum: Migraine aura-like symptoms at onset of stroke and stroke-like symptoms in migraine with aura

**DOI:** 10.3389/fneur.2023.1178144

**Published:** 2023-03-13

**Authors:** Frontiers Production Office

**Affiliations:** Frontiers Media SA, Lausanne, Switzerland

**Keywords:** migraine aura, ischemic stroke, spreading depolarization, differential diagnosis, clinical

An omission to the funding section of the original article was made in error. The following sentence has been added: “Open access funding was provided by the University of Bern.”

The original article has been updated.

