# Supplementary material for: Migraine aura-like symptoms at onset of stroke and stroke-like symptoms in migraine with aura
Source: Front Neurol. 2022 Sep 14;13:1004058. doi: 10.3389/fneur.2022.1004058 (PMC9531679; doi:10.3389/fneur.2022.1004058)
Supplement: Supplementary file 1 [file Data_Sheet_1.pdf]

ID

____

Questionnaire

If Stroke-Wake-Up? □ **yes** □ **no**

1) Have you had any visual disturbances? □ *yes* □ *no. If yes,*

a) Type of visual disturbance?

□ blurred □ bright □ colored □dark/black □ zigzag lines □scintillating scotoma □ other

____

____

b) Localization: □monocular □ binocular homonymous □binocular on totality of visual field

c) Migration of visual disturbances? □ yes □no (were fixed). If yes, please select:

□from center to periphery

if checked: □to the left □to the right □upwards □downwards

□From the periphery to the center

if checked: □ from above □ from below □ from the right □ from the left

____

____

e) Time of onset

□ suddenly □ < 59 seconds □60sec-4 minutes 59 seconds □5-59minutes □60 minutes-24h □>24h □ unclear

f) Duration of the visual disturbance (beginning to end)

□ < 59 seconds □60sec-4 minutes 59 seconds □5-59minutes □60 minutes-24h □>24h □ unclear

2) Have you had any sensory disturbances? □yes □no.

a) Type of sensory disturbance? □tingling/pins and needles □numbness □ other

____

____

b) If yes, where were they located? (please select)

□ left - □ right - □ both sides

□ tongue - □ face - □ arm - □ leg □ torso

c) Have they migrated? □*yes* □*no*

If yes, please specify order (tongue, face, arm, leg, trunk)

____

____

d) Time of onset

□ suddenly □ < 59 seconds □60sec-4 minutes 59 seconds □5-59minutes □60 minutes-24h □>24h □ unclear

e) Duration of the sensory disturbance (beginning to end)

□ < 59 seconds □60sec-4 minutes 59 seconds □5-59minutes □60 minutes-24h □>24h □ unclear

***3) Have you had any weakness?* □yes □no. If yes,**

a) where were they located? (please choose)

□ left - □ right - □ both sides

□ tongue - □ face - □ arm - □ leg - □torso

b) Have they migrated? □*yes* □*no*

If yes, please specify order (tongue, face, arm, leg, trunk)

____

____

____

____

c) Time of onset

□ suddenly □ < 59 seconds □60sec-4 minutes 59 seconds □5-59minutes □60 minutes-24h □>24h □ unclear

d) Duration of the motor disturbance (beginning to end)

□ < 59 seconds □60sec-4 minutes 59 seconds □5-59minutes □60 minutes-24h □>24h □ unclear

4) Have you had difficulty speaking? □yes □no.

If yes

a) of what kind? (please choose)

□difficulty finding the right words □difficulty understanding what others have said □difficulty articulating the words □unfamiliar words spoken □neologisms □others

____

____

b) Time of onset

□ suddenly □ < 59 seconds □60sec-4 minutes 59 seconds □5-59minutes □60 minutes-24h □>24h □ unclear

c) Duration of the speech disturbance (beginning to end)

□ < 59 seconds □60sec-4 minutes 59 seconds □5-59minutes □60 minutes-24h □>24h □ unclear

5) a) What was the order of: Visual disturbances, speech disturbances, sensory disturbances, and weakness? Please describe:

____

____

____

b) Time interval between symptoms? Eg, Sensory->Weakness->Visual disturbance

Symptom 1 _________ ____after _____ minutes

Symptom 2_______________after_______minuets

Symptom 3_______________after_______minutes

Symptom 4_______________after_______minutes

Symptom 5_______________after_______minutes

6) How many episodes with the symptoms described above have you had so far?

____

when did the last episode occur?

____

7) Have you experienced a headache after or at the same time as the symptoms described above?

If yes

- *Did the symptoms* occur □ before □after, or □during the headache?

- *where was the headache located?* ______________________

- *Character* □pulsating pain □throbbing pain □shooting pain □pressing pain □burning

- *how long did it last?* ________________

- *how severe was the pain? (1-10/10)* ______

- *Was the pain related to physical activity?* □Yes □No

if yes □ the pain decreased □the pain increased

- during the headache you had □ nausea, □photophobia, □phonophobia

8) Have you had trouble concentrating? □yes □no.

If so,

Duration (beginning to end)

□ < 59 seconds □60sec-4 minutes 59 seconds □5-59minutes □60 minutes-24h □>24h □ unclear

9) Were you been disoriented? ***□yes □no***

If so,

Duration of disorientation (onset to end)

◻ < 59 seconds □60sec-4 minutes 59 seconds □5-59minutes □60 minutes-24h ◻>24h ◻ unclear

10) Have you had trouble swallowing? ***□yes □no***

If yes

Duration of difficulty swallowing (beginning to end)

◻ < 59 seconds □60sec-4 minutes 59 seconds □5-59minutes □60 minutes-24h ◻>24h ◻ unclear

11) Have you had difficulty walking? ***□yes □no:***

If so,

a) Time of onset

◻ suddenly ◻ < 59 seconds □60sec-4 minutes 59 seconds □5-59minutes □60 minutes-24h ◻>24h ◻ unclear

b) Duration of the difficulty walking (beginning to end)

◻ < 59 seconds □60sec-4 minutes 59 seconds □5-59minutes □60 minutes-24h ◻>24h ◻ unclear

12) Have you experienced trouble coordination in your arms (e.g., when spreading butter on bread) or legs (e.g., when walking)? ***□yes □no.***

If so,

a) where have you felt it?

□ left - □ right - □ both sides

□ Tongue - □ Face - □ Arm - □ Leg - □Torso

a) Time of onset

◻ suddenly ◻ < 59 seconds □60sec-4 minutes 59 seconds □5-59minutes □60 minutes-24h ◻>24h ◻ unclear

b) Duration of the coordination disturbance (beginning to end)

◻ < 59 seconds □60sec-4 minutes 59 seconds □5-59minutes □60 minutes-24h ◻>24h ◻ unclear

13) Have you had vertigo? □yes □no

If so,

a) was this

□rotatory to the right □ rotatory to the left □ swaying

b) Time of onset

◻ suddenly ◻ < 59 seconds □60sec-4 minutes 59 seconds □5-59minutes □60 minutes-24h ◻>24h ◻ unclear

c) Duration of the vertigo (beginning to end)

◻ < 59 seconds □60sec-4 minutes and 59 seconds □5-59minutes □60 minutes-24h ◻>24h ◻ unclear

14) Have you had double vision? □yes □no

...If so,

a) were they □horizontal □vertical □ oblique offset? (please choose)

b) Time of onset

◻ suddenly ◻ < 59 seconds □60sec-4 minutes 59 seconds □5-59minutes □60 minutes-24h ◻>24h ◻ unclear

c) Duration of the double vision(beginning to end)

◻ < 59 seconds □60sec-4 minutes 59 seconds □5-59minutes □60 minutes-24h ◻>24h ◻ unclear

15) Have you had a hearing disturbance? □yes □no

If so,

a) Time of propagation

□ < 59 seconds □60sec-4 minutes 59 seconds □5-59minutes □60 minutes-24h □>24h □ unclear

b) Duration of the hearing disturbance (beginning to end)

□ < 59 seconds □60sec-4 minutes 59 seconds □5-59minutes □60 minutes-24h □>24h □ unclear

16) Cardiovascular risk factors

a. Do you smoke? If yes ______PY
b. Do you drink alcohol? If yes _________Drinks/month
c. Do you take drugs?
d. Have you been diagnosed with high blood pressure? □yes □no
e. Have you been diagnosed with diabetes mellitus? □yes □no
f. Do you have a family history of stroke and/or heart attack? □yes □no. If yes, at what age did these occur? _____
g. Do you have high cholesterol? □yes □no
h. Have you been diagnosed with sleep apnea? □yes □no □unknown
i. How much do you weigh and how tall are you? ____kg_____cm
j. Have you been diagnosed with depression? □yes □no
k. (for women) are you taking birth control pills? □yes □no
l. Have you been diagnosed with renal insufficiency? □yes □no
m. Have you been diagnosed with atrial fibrillation □yes □no, or other cardiac arrhythmias? If yes, which ones? _______
n. Have you been diagnosed with a chronic inflammatory disease? □ yes □ no

17) Varia

Have you been diagnosed with: peripheral arterial disease □, coronary heart disease□
